# Bilaterally primary cementless total hip arthroplasty in patients with ankylosing spondylitis

**DOI:** 10.1186/1471-2474-15-344

**Published:** 2014-10-11

**Authors:** Wanchun Wang, Guoliang Huang, Tianlong Huang, Ren Wu

**Affiliations:** Department of Orthopaedics, The Second Xiangya Hospital, Central South University, Changsha, Hunan 410011 P.R. China

**Keywords:** Total hip arthoplasty, Ankylosing spondylitis, Noncemented

## Abstract

**Background:**

The purpose of this study was to document the clinical and radiographic results of a consecutive series of patients with ankylosing spondylitis (AS) who had undergone bilaterally primary THA using non-cemented components.

**Methods:**

Twenty-six hips of 13 patients with bilateral ankylosis of the hip caused by AS were converted to THA from January 2000 to January 2008. The mean age of the patients was 33.7 years (range, 22-57 years). There were 11 males and 2 females. All the patients had bilateral bony ankylosis with 0° range of motion. The average Harris Hip Scores (HSS) was 22.1 (10–38).

**Results:**

At a mean follow-up of 128.4 months, the mean HSS at the latest follow-up examination was 91.7 points (range, 75-98 points). All the patients reported marked relief of painful symptoms. Three (11.5%) of the 26 hips were outside the Lewinnek acetabular cup inclination safe range, and 5 (19.2%) of the 26 hips were outside the Lewinnek acetabular cup anteversion safe range. The probability of survival of the prostheses was 100% at 5 years and 92.3% at 10 years, but it dropped sharply to 73.1% at 13 years.

**Conclusion:**

Cementless THA is a worthwhile treatment for the osseous ankylosed hip joint caused by AS. Newfound mobility, maneuverability, and improved ability to sit comfortably were the outcomes that alleviated the patients’ daunted morale. However, the technically demanding nature of the procedure should not be underestimated.

**Electronic supplementary material:**

The online version of this article (doi:10.1186/1471-2474-15-344) contains supplementary material, which is available to authorized users.

## Background

Ankylosing spondylitis (AS) affects mainly physically and economically active young men. The typical age of onset of this seronegative spondyloarthropathy is 15 to 25 years of age[1–3]. It is characterized by inflammation of the axial spine, sacroiliac joints, and peripheral joints. Peripheral joints involvement has been reported in 73% of these cases, with the hip being the most commonly affected diarthrodial joint[3, 4]. Among those with the hips affected, which accounts for 25% to 50% of patients with AS, 50% to 90% are involved bilaterally[1–3].

Total hip arthroplasty (THA) is now the well-accepted standard treatment for patients with severe hip involvement[1–8]. The purpose of this study was to document the clinical and radiographic results of a consecutive series of patients with AS who had undergone bilaterally primary THA using non-cemented components.

## Methods

Twenty-six hips of 13 patients with bilateral ankylosis of the hip caused by AS were converted to THA by a single surgeon from January 2000 to January 2008 at our institution. The mean age of the patients was 33.7 years (range, 22–57 years). There were 11 males and 2 females (see Table 1). The diagnosis of AS was confirmed in all the patients according to the New York criteria[9]. In no patient was there evidence of any other seronegative spondyloarthropathy. Ankylosis of the hip was defined by physical examination as a total loss of hip motion. Only bony ankylosis cases were included. All the patients had bilateral bony ankylosis with 0° range of motion (ROM). The average Harris Hip Scores (HSS)[10] was 22.1 (10–38). This study was approved by the Second Xiangya Hospital committee for clinical research and informed consent was obtained from the patients participating in the study. The patients provided written informed consent for the publication of individual clinical details and accompanying images.

**Table 1:** **Patients’ data**

Case	Side	Age/sex	Pre-op HSS (PS)	Initial position of the limb		Inclination of the cup	Anteversion of the cup	Latest HSS(PS)	Revisions (months)	Follow-up (months)
				Abduction	Abduction					
1	Right	32/Male	24(20)	10°	15°	45.5°	15.9°	88(40)	1(149)	170
	Left		24(20)	10°	25°	43.0°	10.5°	92(44)	1(155)	170
2	Right	27/Male	35(30)	15°	35°	35.5°	15.5°	98(44)		165
	Left		35(30)	20°	15°	41.3°	10.7°	98(44)		165
3	Right	36/Male	28(20)	10°	25°	44.6°	12.5°	93(44)	1(141)	161
	Left		28(20)	15°	20°	48.9°	16.7°	93(44)	1(135)	161
4	Right	29/Male	24(20)	20°	40°	*53.2°	9.6°	95(44)		155
	Left		24(20)	20°	35°	46.3°	*0°	95(44)		155
5	Right	45/Female	10(10)	10°	25°	42.5°	7.5°	88(40)	1(137)	150
	Left		10(10)	15°	20°	43.7°	17.5°	88(40)	1(116)	150
6	Right	22/Male	25(20)	20°	40°	46.9°	6.3°	98(44)		143
	Left		25(20)	15°	30°	42.1°	*0°	98(44)		143
7	Right	37/Male	10(10)	25°	25°	39.5°	7.1°	85(40)		130
	Left		10(10)	10°	30°	46.4°	15.7°	85(40)	1(103)	130
8	Right	49/Male	10(10)	15°	25°	36.7°	*26.1°	91(44)		121
	Left		10(10)	15°	25°	33.2°	16.7°	91(44)		121
9	Right	28/Male	38(30)	10°	15°	38.5°	22.3°	98(44)		117
	Left		38(30)	10°	30°	39.1°	11.5°	98(44)		117
10	Right	57/Female	24(20)	35°	40°	*51.5°	13.7°	75(30)		106
	Left		24(20)	30°	40°	49.5°	*0°	75(30)		106
11	Right	41/Male	24(20)	15°	50°	48.5°	12.7°	91(44)		96
	Left		24(20)	20°	60°	*56.3	19.3°	91(44)		96
12	Right	35/Male	10(10)	5°	25°	36.8°	13.2°	95(44)		82
	Left		10(10)	10°	15°	33.7°	8.5°	95(44)		82
13	Right	53/Male	25(20)	15°	45°	39.5°	*0°	98(44)		73
	Left		25(20)	20°	50°	35.6°	13.4°	98(44)		73

HSS: Harris Hip Score.

PS: Pain Score.

*: Outside the safe range proposed by Lewinnek et al.[16].

The indication for surgery was progressive disabling pain in the low back or knee, loss of function caused by immobility or malposition of both hips, severe limping and walking disability[3, 4]. Contraction of the abductor muscle was palpable in all hips in this study. Patients without functioning abductor muscle were not indicated for conversion of their hip arthrodesis to THA.

### Surgical technique

Preoperatively, X-ray templating was undertaken to carefully plan anatomical reconstruction and assess the use of the most appropriate approach depending on patient anatomy. Preoperative parenteral antibiotics and prophylaxis for deep vein thrombosis was used in all patients. All patients received bilateral THA in a single session. Based on the deformity, the worst hip was chosen to replace first.

**Figure 1:**
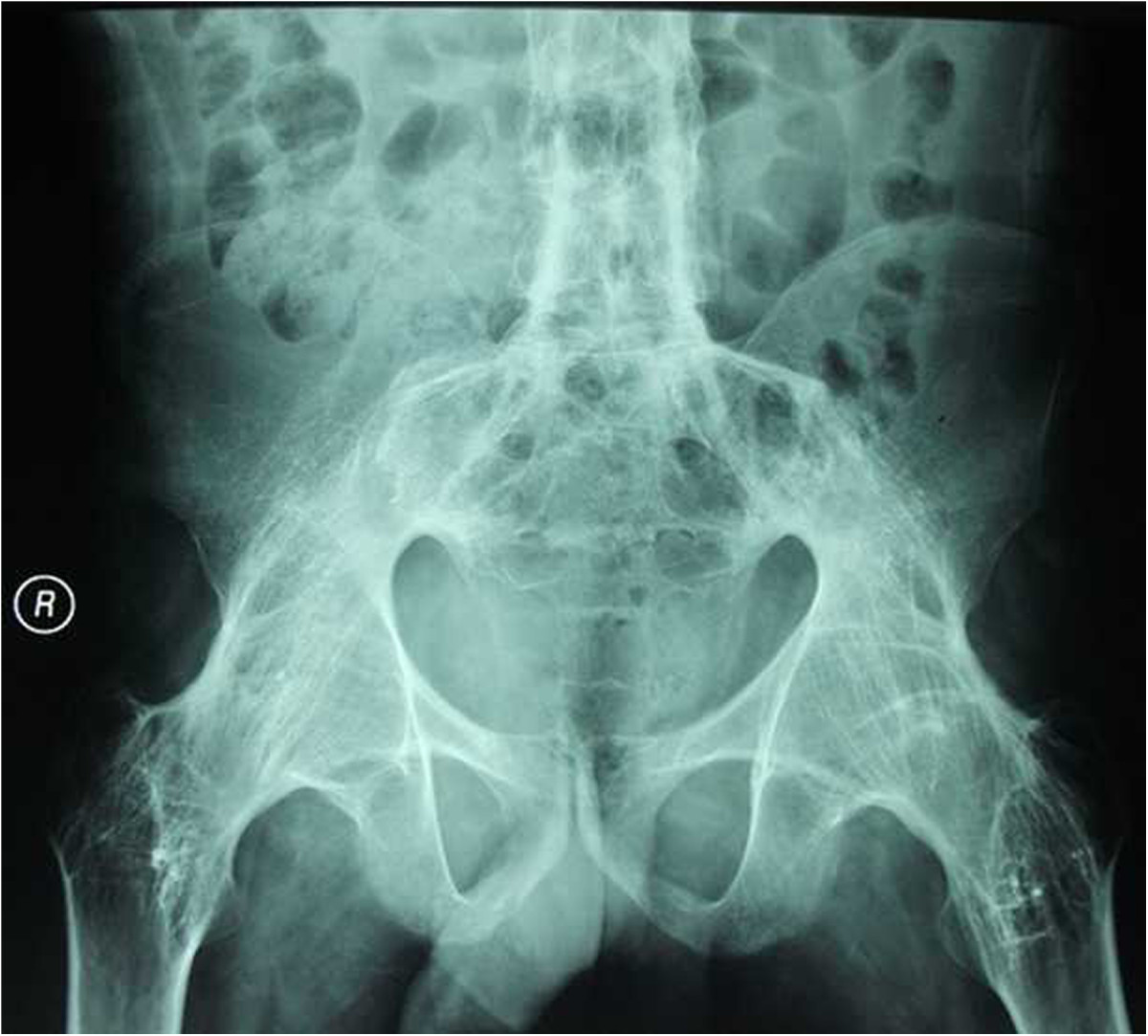
**The radiographs of a 35-year-old male with AS was shown.** The patient had bilateral bony ankylosis with 0° range of motion.

**Figure 2:**
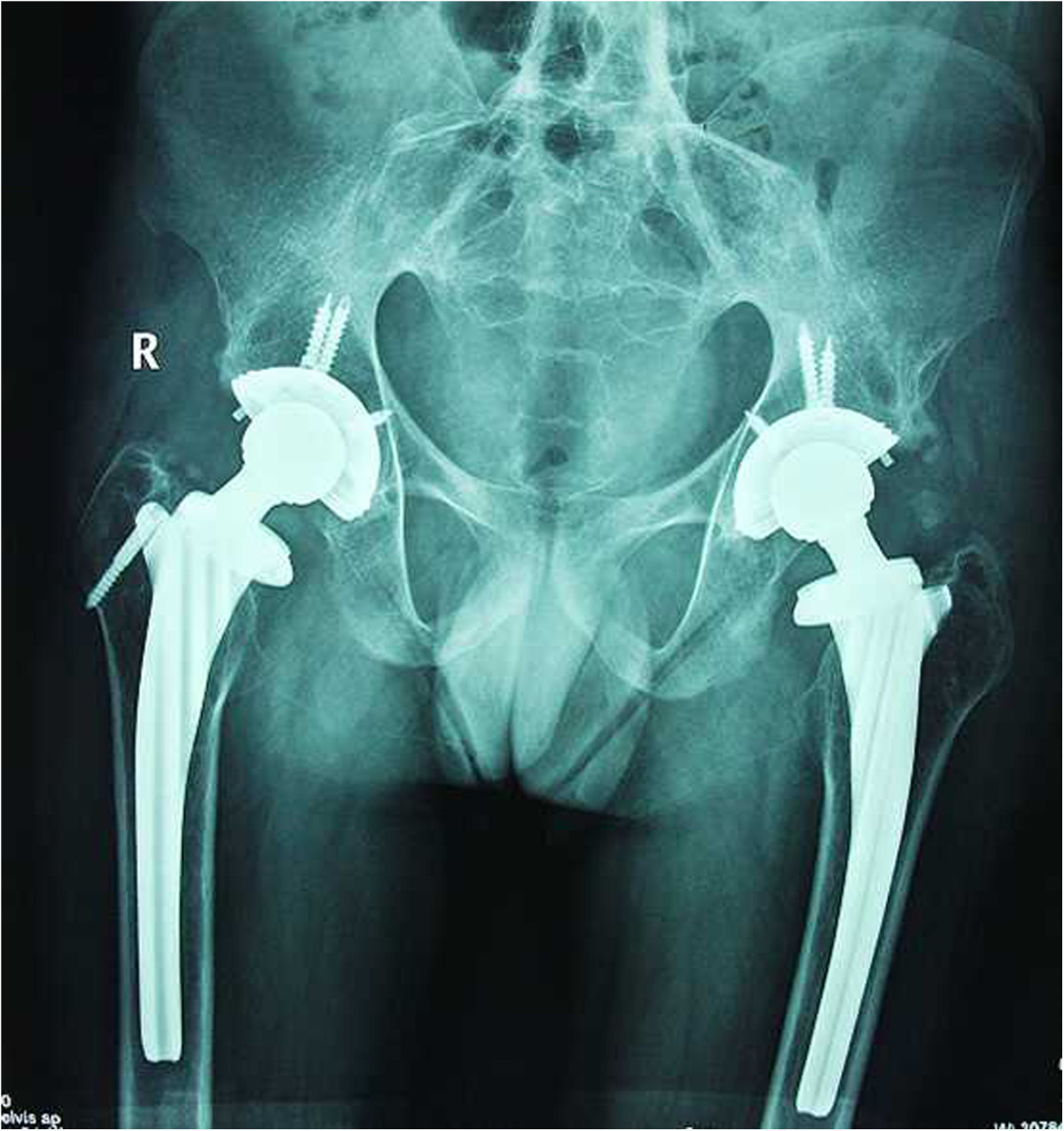
**Postoperative radiograph of the patient two weeks after cementless THA.**

Operations were carried out in the lateral position for all hips by using a standard transtrochanteric approach. After detaching the external rotators, the femoral neck resection line was identified by approaching the inferior neck and locating the pubofemoral arch and lesser trochanter. Using a two-step system, at an angle of 45° at the juncture of the femoral head and neck, the femoral neck was cut. Secondly, the joint was dislocated and the remaining portion of the femoral neck removed[11]. The articular capsule and osteophytes were thoroughly excised to sufficiently expose the acetabulum. No trochanteric osteotomy was performed. Acetabular preparation started with removal of the remaining femoral-head piecemeal. Reaming with sequentially larger reamers was then done in the medial direction, as it is difficult to identify the location of the original joint plane and ensure optimum positioning of components. In our study, we used the foveal soft tissue as an indication for locating the original joint plane. Although hips had bony fusion, there remained incomplete gray ossifying cartilage at the location of the original joint plane, which also helped to identify the location of the original joint plane. In some instances, intraoperative radiographs were taken to identify the original joint plane, which may have been useful. We used a transverse acetabular ligament as a guide to appropriate acetabular prosthesis anteversion and the lesser trochanter as a guide to appropriate femoral prosthesis anteversion. According to the specific hip deformity, we determined the prosthesis insertion angle and performed a trial reduction to check stability in all directions, correction of the fixed deformity and limb length. After this trial reduction, definitive components were implanted[11]. In all hips, short external rotator muscles were closed after the surgical procedure and the wound closed with a drain. All patients received uncemented THAs (Link, Germany) (see Figures 1,2 and3).

**Figure 3:**
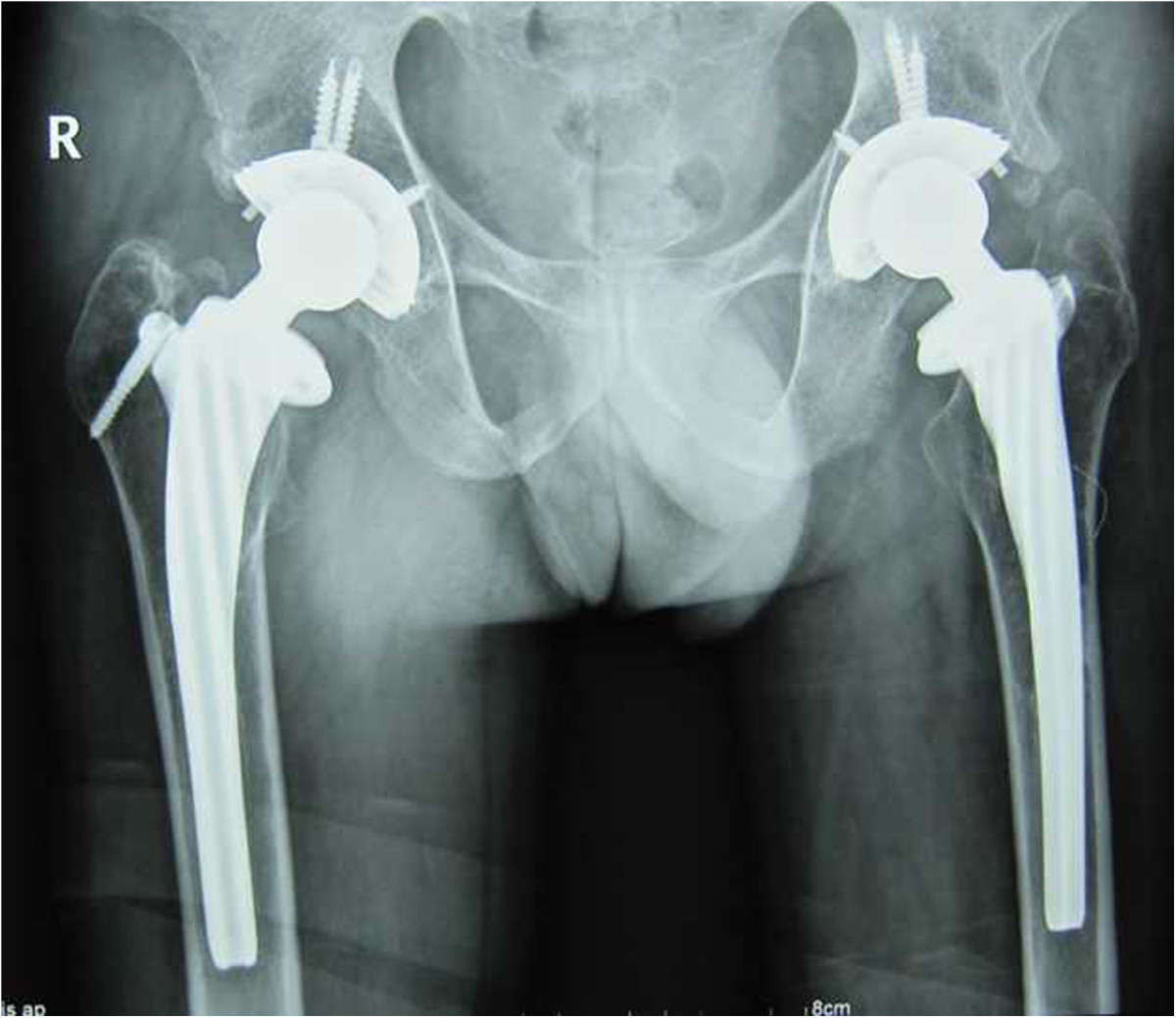
**Postoperative radiograph of the patient 7 years after cementless THA.**

Postoperative care: Prophylactic anti-inflammatory drug (Celebrex, 200 mg, QD) was used to prevent heterotopic ossification. Physiotherapy started on the second post-operative day. Patients were usually allowed to walk with support after three days, and full weight bearing was permitted after at least two weeks.

Patients were evaluated clinically using both subjective and objective information based on the HSS. Postoperative evaluation of patients was performed at six weeks, three months, six months, one year, and then annually. Radiographic evaluation was also performed at each clinical interval. Cementless stem loosening was defined according to the criteria of Engh et al.[12] Heterotopic ossification was classified using criteria of Brooker et al.[13].

Acetabular cup inclinations were measured directly on AP radiographs[14]. First, a horizontal line connecting both teardrops was drawn, and then a line was drawn through the longest diameter of the elliptical opening of the acetabular cup rim. The angle between these two lines was recorded as cup inclination. In cases with a poor teardrop outline or an asymmetric pelvis, a vertical line was drawn bisecting the sacrum and a line perpendicular to this line was used as reference horizontal line for measurement purposes. The method proposed by Pradhan[15] was used to calculate acetabular cup anteversion in AP radiographs. Acetabular cup inclination and anteversion were classified as to whether they were in or out of the safe range (40° ±10° for inclination and 15° ±10° for anteversion) proposed by Lewinnek et al.[16].

## Results

At a mean follow-up of 128.4 months (range, 73-170 months), the mean HSS at the latest follow-up examination was 91.7 points (range, 75–98 points).

**Figure 4:**
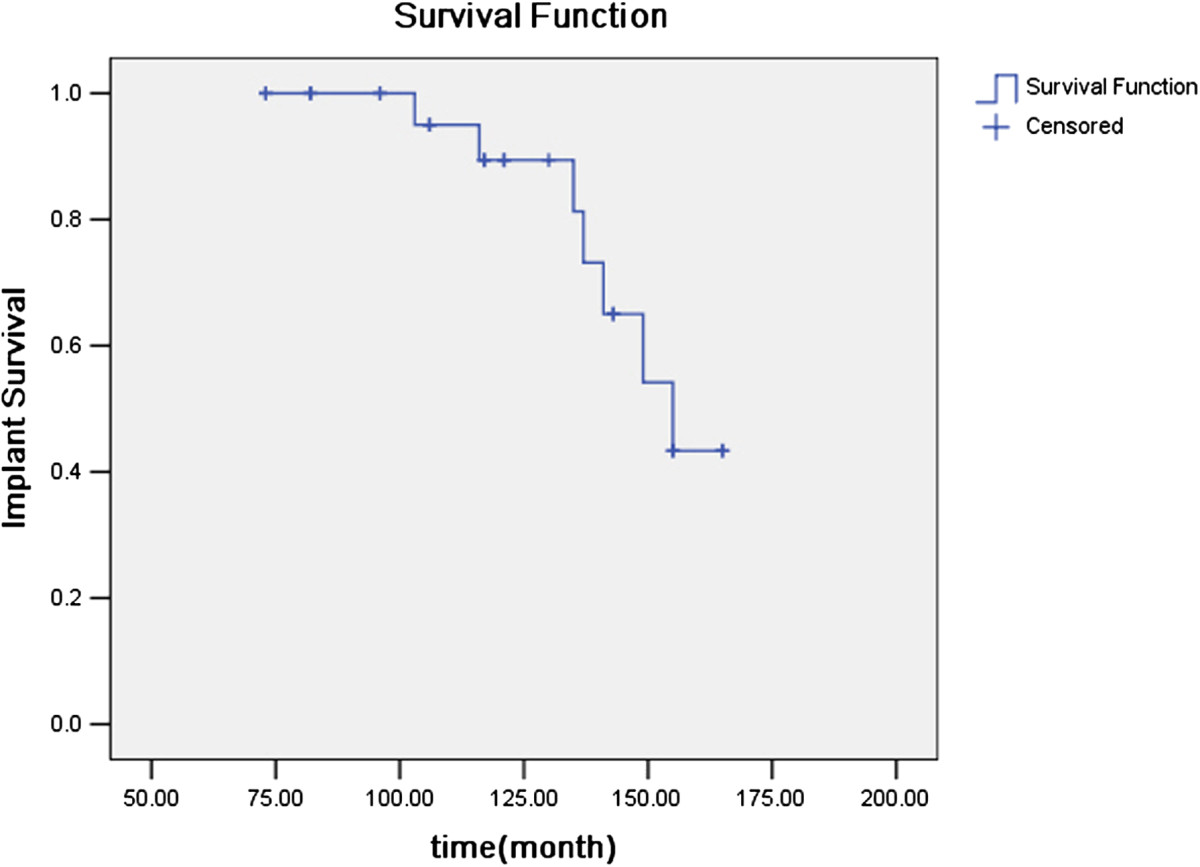
**Kaplan-Meier implant survival curve analysis for THA with revision surgery as end point.**

All the patients reported marked relief of painful symptoms. Before surgery, the mean pain score was 18.5 points (range, 10-30 points) of a possible 44 points, with patients reporting moderate or marked pain in 22 of 26 (84.6%) affected hips. At the latest follow-up, the mean pain score was 42.2 points (range, 30-44 points), with 24 hips (92.3%) having slightly or no pain in the affected hip. Preoperative positions of fused hips are detailed in Table 1.Osteolysis due to polyethylene wear was found in 3 hips (out of the 3 hips, one patient with two hips). The patient with bilateral osteolysis at the acetabular and femoral sides underwent polyethylene liner exchange with bone grafting of the osteolytic lesions bilaterally. The left hip was operated on 116 months and the right side was operated on 137 months after index operations. The other patient had osteolysis at the acetabular side in the left hip with cup loosening and was revised 103 months after index operations. Radiographic evidence of cup and stem loosening was detected in 2 patients bilaterally. For one patient, the left hip was revised 135 months after the index operation and the right side was revised 141 months after index operation. For the other patient, the right hip was revised 149 months after the index operation and the left side was revised 155 months after index operation. The probability of survival of the prostheses was 100% at 5 years and 92.3% at 10 years, but it dropped sharply to 73.1% at 13 years (see Figure 4).

Local complications associated with the procedure include 1 (3.8%) postoperative peroneal nerve palsies, which had complete return of function nearly 6 months following the procedure. Two patients (7.7%) sustained intraoperative nondisplaced fracture at the level of the calcar. One patient (3.8%) had femoral fracture during stem implantation, and circumferential wiring was required. All of these intraoperative fracture healed uneventfully. There was no infection or postoperative dislocation.

Overall inclinations and anteversions of acetabular cups are shown in Table 1. Three (11.5%) of the 26 hips were outside the Lewinnek acetabular cup inclination safe range, and 5 (19.2%) of the 26 hips were outside the Lewinnek acetabular cup anteversion safe range. Overall cup malposition, whether in anteversion or inclination, was observed in 8 (30.7%) of the 26 hips.

At the latest follow-up examination, heterotopic ossification was present in 5 of 26 hips (19.2%) which had perioperative prophylaxis for heterotopic bone formation. All five hips with radiographic evidence of heterotopic ossification were Brooker classes I and II, which were not given any advanced treatment.

## Discussion

Results of this investigation suggest that THA using noncemented techniques provides an acceptable outcome at follow-up periods of 73–170 months. The average Harris hip scores improved from 22.1 points before surgery to 91.7 points at a mean follow-up of 128.4 months, and the probability of survival of the prostheses was 100% at 5 years and 92.3% at 10 years. This compared favorably with the series of AS cases of Tang and Chiu[3], who reported the probability of survival of the noncemented prostheses was 95.5% at both the 5-year and 10-year intervals, but it dropped sharply to 63.6% at 11 years. Kim et al.[14] reported 24 ankylosed hips were converted to THAs in 12 patients. Osteolysis and loosening were found in 3 and 2 hips, respectively. Joshi et al.[5] reported a large series about THA for patients with AS. Their study involved 181 hips with a follow-up of 27 years. Survivorship analysis showed survival of the implant was 87.3% at 10 years. However, only 65% had excellent hips function in their study. Ye et al.[16] reported 15 AS patients with bilateral hip ankylosis were managed with cementless bilateral synchronous THA and revealed that cementless bilateral synchronous THA is a safe and effective approach in the treatment of AS with bilateral hip ankylosis. However, their mean follow-up was only 29.3 months.

In this study, the rate of malposition of the acetabular cup was 30.7% according to the safe range defined by Lewinnek[17], which is lower than the rate of other studies[3, 11, 14]. The direction of the malpositioning showed a tendency to be defined by the fixed position of the contralateral limb[3, 11]. Kim et al.[14] found that if abduction or adduction of the contralateral limb is combined with fixed flexion contracture, the pelvis will tilt in the axial plain. Fixed adduction and flexion tilts the pelvis anteriorward and creates the possibility of inserting the cup with less anteversion than desired. Fixed abduction and flexion presents the opposite possibility, i.e., of inserting the cup with more anteversion than desired. In our study, three-dimensional computed tomography and intraoperative radiograph were taken, which were useful for inserting an acetabular cup in a good position. We agree with the methods proposed by Banjian et al.[11]. First, inserting the prosthesis as a routine THA when it was a simple flexion deformity; if combined with limb internal rotation, the anteversion angle of the acetabular cup was reduced and the anteversion angle of the femoral prosthesis was increased. In contrast, the anteversion angle of the acetabular cup was increased and the anteversion angle of the femoral prosthesis was reduced or kept the anteversion angle of the femoral prosthesis at 0° when it was combined with external rotation deformity of the limb. When combined with hip adduction deformity, some contracted adductors were removed and the acetabular cup inclination angle was reduced, which may reduce hip function but will enhance stability.

Malpositioning of an acetabular cup increases the risk of postoperative dislocation. In our study, there was no postoperative dislocation even in the cases in which the acetabular cup was inserted out of the safe range. Malpositioned components seemed to be well tolerated in these cases. There was no evidence that a malpositioned cup increased the polyethylene wear, osteolysis, or implant loosening.

High rates of heterotopic ossification have been reported in the patients with AS undergoing THA[3, 4, 18]. The reported incidence varied widely[3, 4, 18]. In Brinker et al.’s[3] report, heterotopic ossification was observed in 6 of 14 hips (43%) in the patients with AS who had not had any perioperative prophylaxia. In the study reported by Tang and Chiu[4], 21.0% of arthroplasties developed Brooker class III and IV heterotopic ossification. In their study, prophylactic treatment for ectopic ossification was not routinely given. In our study, heterotopic ossification (Brooker classes I and II) was present in 5 of 26 hips (19.2%) which had perioperative prophylaxis for heterotopic bone formation. There was no Brooker class III and IV heterotopic ossification. Indomethacin prophylaxis for 2 weeks' duration was selected because it is cost-effective, and the side effects with short duration of prophylaxis are minimum.

## Conclusion

Cementless THA is a worthwhile treatment for the osseous ankylosed hip joint caused by AS. Newfound mobility, maneuverability, and improved ability to sit comfortably were the outcomes that alleviated the patients’ daunted morale. However, the technically demanding nature of the procedure should not be underestimated.

## Authors’ original submitted files for images

Below are the links to the authors’ original submitted files for images. Authors’ original file for figure 1 Authors’ original file for figure 2 Authors’ original file for figure 3 Authors’ original file for figure 4
