# Supplementary figures and images for: Bilaterally primary cementless total hip arthroplasty in patients with ankylosing spondylitis
Source: BMC Musculoskelet Disord. 2014 Oct 11;15:344. doi: 10.1186/1471-2474-15-344 (PMC4198795; doi:10.1186/1471-2474-15-344)

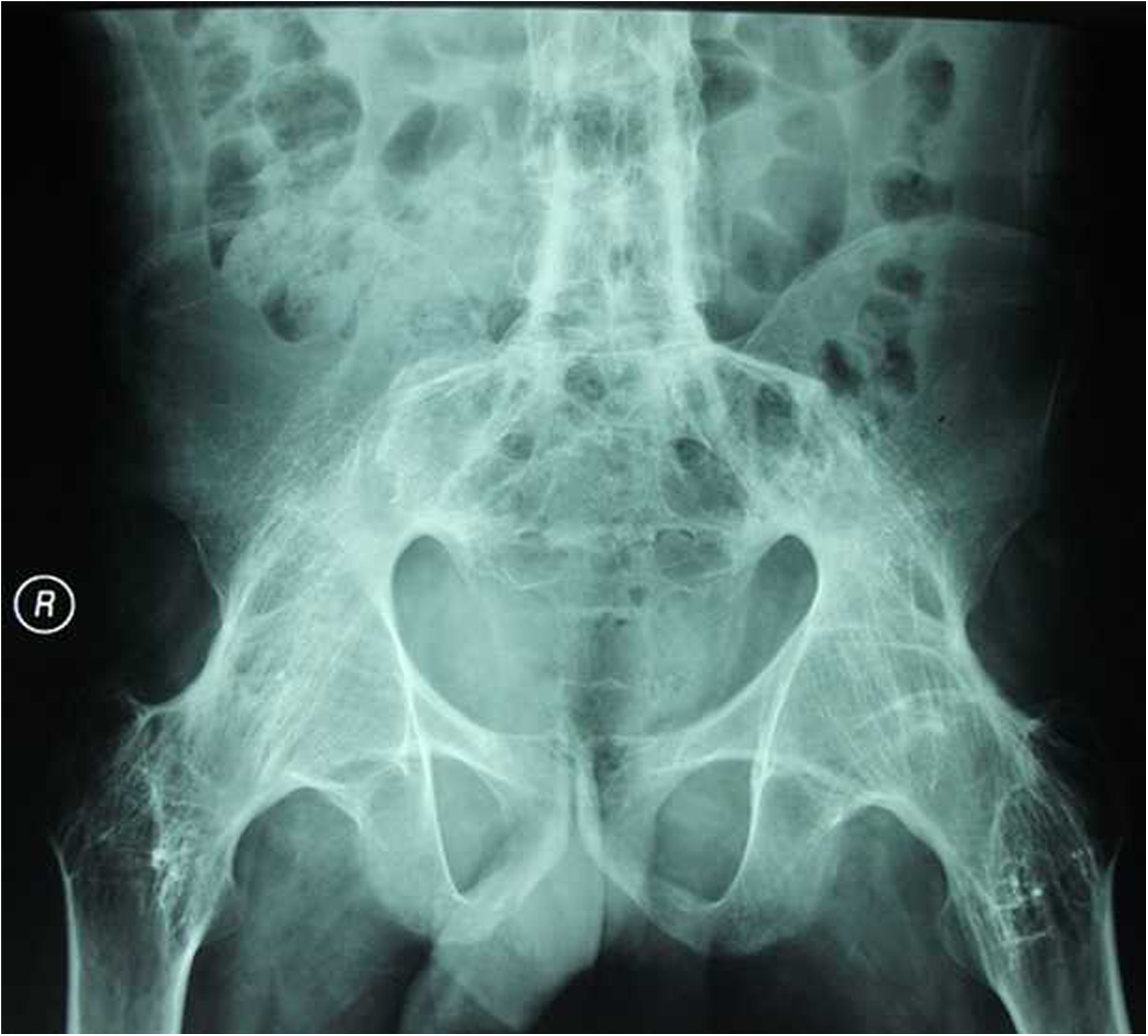

Supplement: Supplementary file 1 — Authors’ original file for figure 1 [file 12891_2014_2276_MOESM1_ESM.tif]

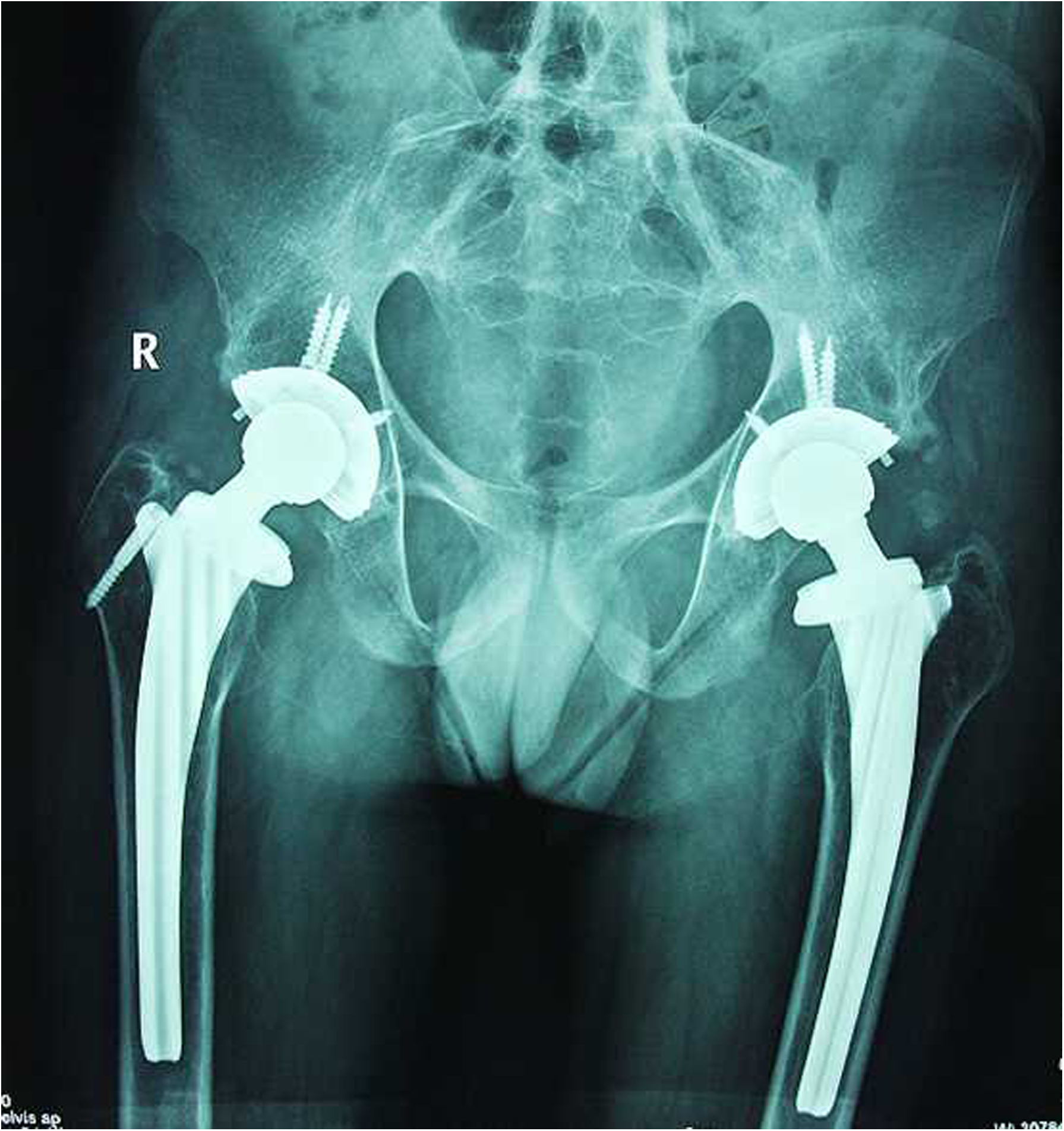

Supplement: Supplementary file 2 — Authors’ original file for figure 2 [file 12891_2014_2276_MOESM2_ESM.tif]

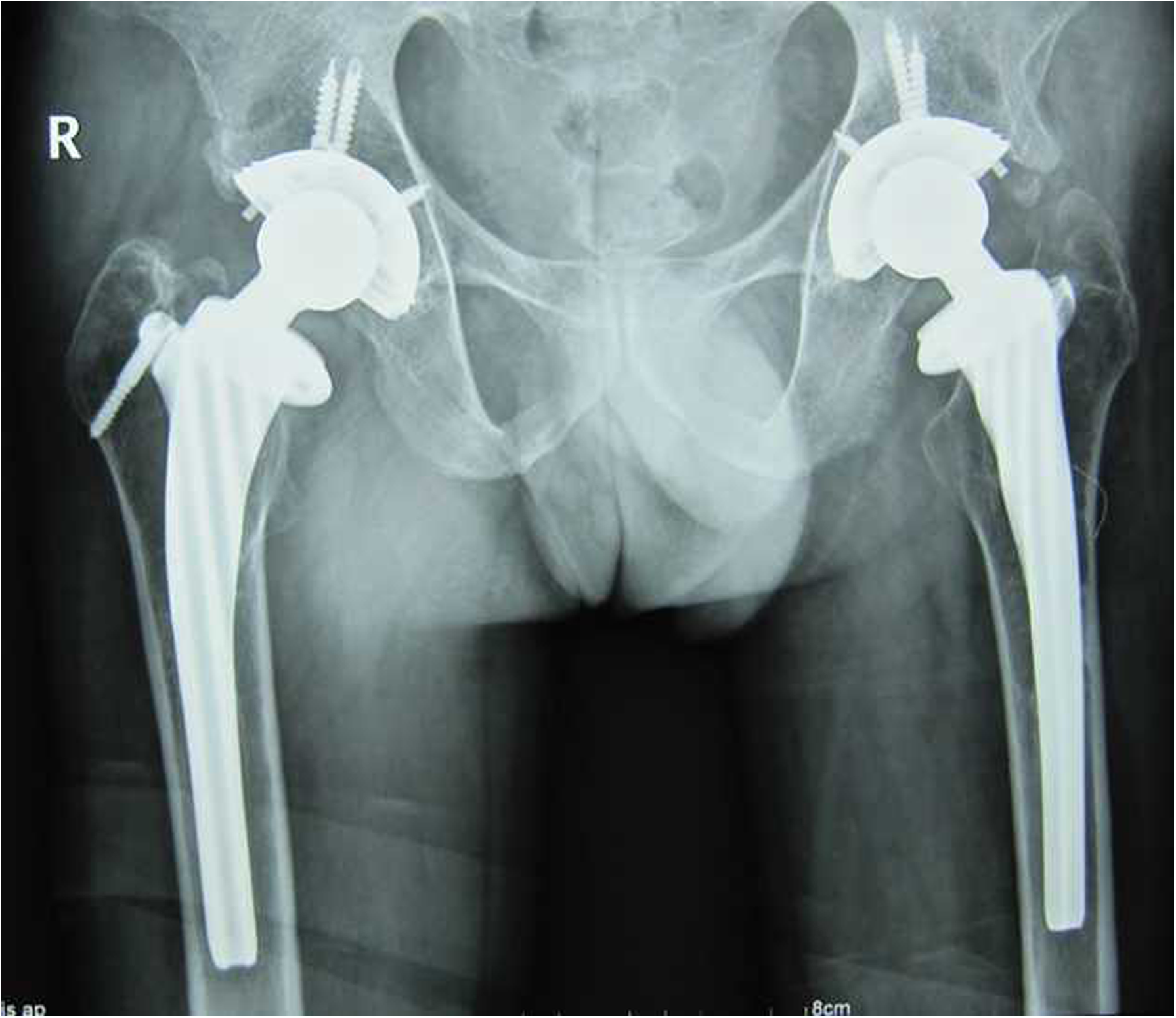

Supplement: Supplementary file 3 — Authors’ original file for figure 3 [file 12891_2014_2276_MOESM3_ESM.tif]

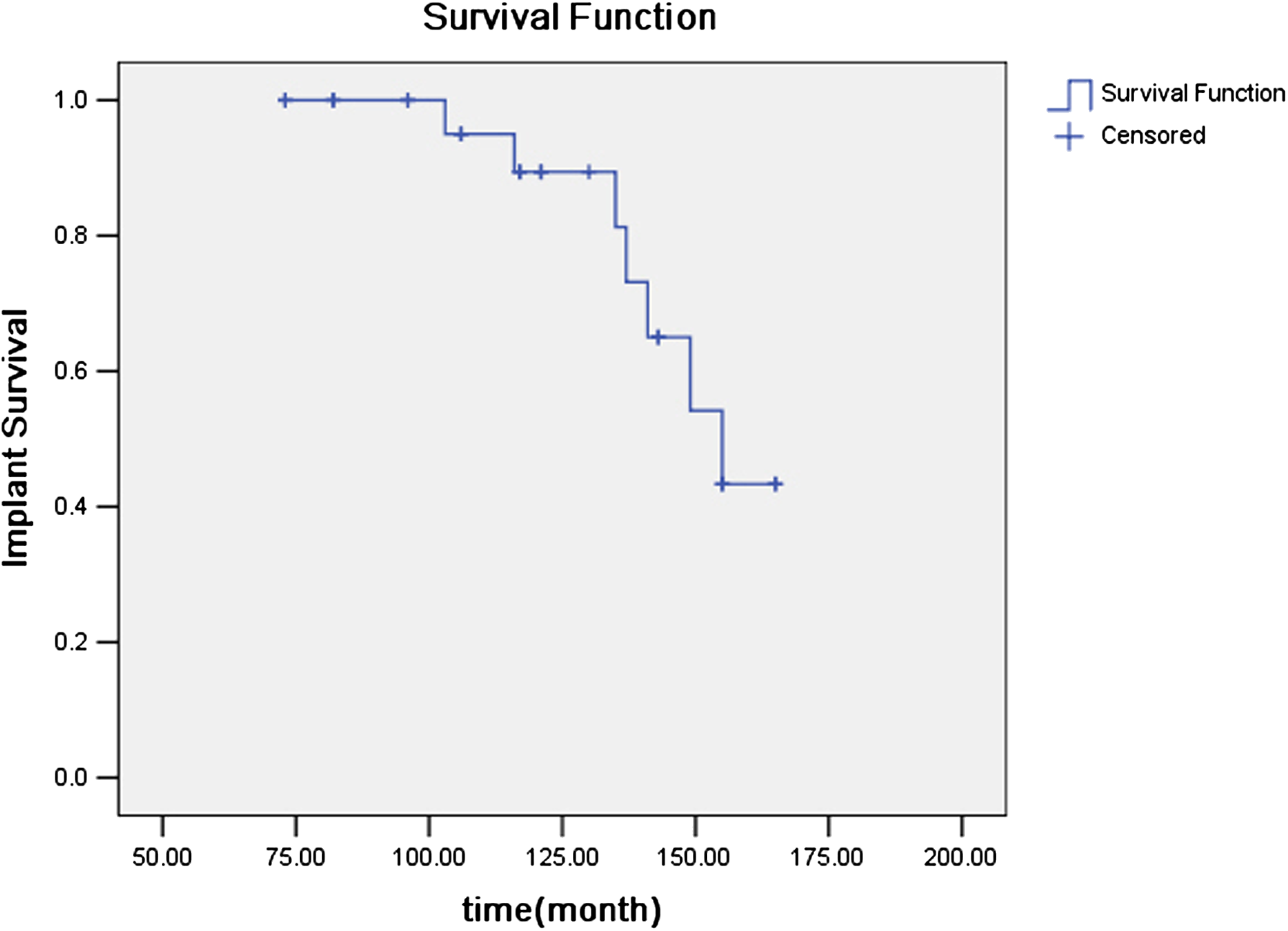

Supplement: Supplementary file 4 — Authors’ original file for figure 4 [file 12891_2014_2276_MOESM4_ESM.tif]
